# A Conjugated Microporous Polymer/Wood Aerogel with Physical Adsorption, Chemical Degradation and Antibacterial Self-Cleaning Triple Sewage Treatment Functions

**DOI:** 10.3390/polym15193929

**Published:** 2023-09-28

**Authors:** Fanwei Kong, Junkang Ge, Zihao Zhu, Chunxia Chen, Jinsong Peng, Xiaobai Li, Bin Li, Hongwei Ma

**Affiliations:** College of Chemistry Chemical Engineering and Resource Utilization, Northeast Forestry University, Harbin 150040, China; m15662556965@163.com (F.K.); junkangge@mail.ustc.edu.cn (J.G.); zzh1997lt@163.com (Z.Z.); ccx1759@163.com (C.C.); jspeng1998@163.com (J.P.)

**Keywords:** conjugated microporous polymers, wood aerogel, physical adsorption, photocatalysis, bacteriostat

## Abstract

Conjugated microporous polymers (CMPs) have important applications in the fields of optoelectronics and sewage treatment due to their high specific surface area, broad visible absorption, processability and simple synthesis process. Biocompatibility, recycling, mass production and solar photodegradation are particularly important in wastewater treatment. Here, A CMP with a high specific surface area and a hierarchical pore structure (CPOP) was constructed based on 4,4′,4″-Tris(carbazol-9-yl)-triphenylamine (3CZ-TPA). Furthermore, a CMP-loaded wood aerogel (CPOP/wood aerogel) with physical adsorption, chemical degradation, bacterial inhibition and self-cleaning properties was prepared by in situ polymerization and used for wastewater treatment. The obtained CPOP/wood aerogel is highly biocompatible and easy to recycle. In addition, the inherent broad visible light absorption property of CPOP endows it with promising photocatalytic properties. Subsequently, we investigated the photocatalytic mechanism of CPOP, and the results showed that it was mainly affected by peroxyl radicals, which implied and confirmed its microbial self-cleaning for secondary cleaning of water pollutants. The reported studies on CPOP/wood aerogel provide a new direction for water purification materials with excellent adsorption, degradation and antibacterial properties.

## 1. Introduction

As the global population and industrialization continue to grow, chemical dyes are used in a wide range of manufacturing industries (e.g., cosmetics, textiles, food and pharmaceuticals), which creates great value but also many environmental problems as a result [1,2,3]. Due to their toxicity, chemical stability, resistance to degradation and significant waste output on a large scale (more than 7 × 10^5^ tons per year, with approximately 10–15% discharged into the water supply), chemical dyes are considered as one of the major contributors to water pollutants [4]. Biological teratogenicity, carcinogenicity and eutrophication of water bodies caused by chemical dye wastes are serious threats to human health, fisheries and other productive activities [5]. Consequently, the treatment of water pollutants containing chemical dyes is currently garnering significant attention from the global scientific community. Among the many developed water pollutants treatment technologies, adsorption is one of the most cost-effective and efficient techniques for the removal of organic dyes from aqueous solutions [6,7,8]. However, conventional porous adsorbents only adsorb organically polluted dyes without degrading them, leading to problems of regeneration and secondary pollution [9]. In addition, microbial or biological imbalances caused by eutrophication of water bodies lead to biological pollution problems. The vast reservoir of organic dye water pollutants necessitates higher demands on the preparation process, environmental requirements for application and recycling in practical applications. Therefore, there is an urgent need to develop novel materials for solving the problem of water pollutants treatment.

Due to their high specific surface area, high biocompatibility, molecular engineering and controllable regulation, conjugated microporous polymers (CMPs) have attracted extensive attention as potential materials for photovoltaics, water pollution control, semiconductors and energy storage [10,11,12,13]. CMPs have unique topological properties and functions, large surface area, high chemical stability and tunable pores; they receive and transmit light energy through delocalized backbones. Due to their high photonic activity and extended conjugation, CMPs are attractive materials for adsorption and photodegradation of organic dyes in aqueous solutions [14,15]. However, most CMPs have certain requirements on the action environment [16,17]. For example, Cai needs to be in the presence of hydrogen peroxide and Li needs ultraviolet light to stimulate catalysis [17,18]. In addition, conjugated microporous polymers are usually in the form of insoluble and immiscible solid powders. Although this improves the stability and biocompatibility of the material, it is always accompanied by the intractable problems of processability and recycling that come with their own nature [19]. Therefore, the development of novel environmentally friendly CMPs for the adsorption and degradation of organic dyes in aqueous solution still needs more exploration.

As mentioned above, CMPs have the advantages of low toxicity and easy preparation, and they have high potential value in water pollution treatment [20,21]. The application of CMPs in water treatment is limited due to challenges associated with poor dispersion and separation, which restrict their effectiveness. To overcome these defects, CMPs need to be anchored to a suitable substrate. Aerogels have been reported to serve as anchoring substrates for CMPs [22,23,24]. Nonetheless, the entire process of building aerogels from raw materials via a top-down approach is usually energy-intensive and time-intensive. Nowadays, there are few reports on the construction of composites for water treatment by in situ growth of CMPs on wood aerogels. Wood aerogel is a low-density, recyclable, biodegradable, highly biocompatible and highly compressible cellulose aerogel with many interconnected arch-shaped substructures [25,26]. It is typically produced from lightweight, highly porous, thin-walled balsa blocks by a top-down method. It can be seen that wood aerogel can be a good carrier for CMPs.

Here, microporous conjugated polycarbazole (CPOP) with permanent porosity was synthesized using 4,4′,4″-Tris(carbazol-9-yl)-triphenylamine (3CZ-TPA) containing a carbazole conjugated core structure as the building unit. The process is straightforward and the yield is high, which provides conditions for large-scale production. CPOP achieves excellent adsorption performance with a high specific surface area, and the introduction of heteroatoms allows CPOP to possess wide-visible absorption, enabling photocatalytic performance under sunlight. It improves the photocatalysis environment and the process can be carried out only in the presence of sunlight, thus promoting its practical applications. Then, the mechanism of photocatalytic degradation of CPOP was analyzed and determined to be affected by peroxyl radicals, and the antibacterial properties of CPOP were deduced and subsequently confirmed. This indicates that CPOP has the purification function to treat microorganisms in water pollutants. Further, wood’s natural porous skeleton was used to prepare wood aerogel as the anchoring matrix of CPOP, which can improve the processability and cycle stability of CPOP on the basis of ensuring excellent sewage treatment performance [27]. The comparison of this study with the data previously reported in the literature is shown in Table S1. The adsorption and photocatalytic properties of CPOP are significantly higher than for most other materials. In addition, CPOP lignin aerogel also has better recycling capabilities than other materials. At the same time, biomass preparation reduces the cost of organic dye treatment agents and provides new ideas and insights for treating organic dyes in wastewater.

## 2. Materials and Methods

### 2.1. Materials and Reagents

Toluene, acetic acid (CH_3_COOH), cuprous iodide (CuI) and sodium chlorite were purchased from Kermel (Tianjin, China). N, N-Dimethylformamide (DMF), anhydrous magnesium sulfate, dichloromethane (CH_2_Cl_2_) and methanol (CH_3_OH) were obtained from Fuyu (Tianjin, China). Tetrahydrofuran, anhydrous ferric chloride, absolute ethanol, rhodamine B (RhB) and potassium bromide (KBr) were purchased from J&K Scientific (Beijing, China). Tris(4-bromophenyl)amine (98%), carbazole (97%), 1,10-phenanthroline (99%) and potassium carbonate (99.9%) were sourced by Xianding (Shanghai, China). All other reagents utilized in the experiment were of analytical grade.

### 2.2. Instruments

The ^1^H NMR was recorded on a Bruker AVANCE III HD spectrometer at 500 MHz (Bruker, MA, USA), using CDCl_3_ as the solvent at 298 K. TMS was used for ^1^H NMR spectra as an internal standard. The MALDI-TOF-MS mass spectra were recorded using an AXIMA-CFRTM plus instrument (Shimadzu, Kyoto, Japan). UV-vis absorption spectra were recorded on an Agilent Cary100 spectrophotometer (Agilent, CA, USA). Fluorescent spectra were measured with a Shimadzu RF-6000 (Shimadzu, Kyoto, Japan). The absolute quantum efficiency was measured by a HORIBA QM8000 spectrometer (HORIBA, Shanghai, China). FT-IR spectra were recorded on a PerkinElmer Spectrum 400 infrared spectrometer (PerkinElmer, MA, USA). The morphology of the fluorescent cellulose films was photographed on a transmission electron microscope (JEOL JEM-2100, Tokyo, Japan).

### 2.3. Preparation of Materials

#### 2.3.1. Synthesis of 3CZ-TPA

The monomer 3CZ-TPA was prepared as follows (Scheme S1). Tris(4-bromophenyl) amine (1.0 g), 1.33 g of carbazole, 0.29 g of 1,10-phenanthroline, 2.2 g of potassium carbonate and 40 mL of DMF were added to a 100 mL double-necked flask (among them, the molar ratio of reactants tris(4-bromophenyl) amine and carbazole was 1:4). Finally, 0.15 g of CuI was added, and the reaction was heated and stirred under nitrogen protection to 160 °C and refluxed for 72 h. After completion of the reaction, it was cooled at room temperature, 15 mL of dichloromethane was added to dissolve the precipitate, the filtrate was obtained by filtration and methanol was added to form a crude product. Afterwards, it was washed with methanol and water 3 times, and the solid obtained was recrystallized in tetrahydrofuran, filtered, and dried to obtain 0.7891 g of off-white solid with a yield of about 78%.

#### 2.3.2. Synthesis of CPOP

The CPOP was prepared as follows (Scheme S1). Anhydrous ferric chloride (1.6 g) and 40 mL of dichloromethane were added to a 250 mL double-necked flask and thoroughly stirred to disperse. After 0.305 g of 3CZ-TPA was added to 68 mL of methylene chloride to form a solution, the 3CZ-TPA solution was added drop by drop into the anhydrous ferric trichloride dispersion, and the reaction was stirred under nitrogen at 25 °C for 24 h (among them, the molar ratio of reactants 3CZ-TPA and ferric chloride was 1:24). After the reaction, a certain amount of methanol was added to precipitate the solid, it was filtered and then the solid was extracted by Soxhlet with methanol and tetrahydrofuran for 24 h. The solid mass was 0.7763 g and the yield was about 77% after drying [28].

#### 2.3.3. Synthesis of CPOP/Wood Aerogel

First, the dried balsam wood (1 × 1 × 1 cm) was put into a mixed solution of toluene and ethanol (toluene: ethanol = 2:1, *v*/*v*) for dewaxing treatment. The dewaxed balsam wood was added to a mixed solution of 5 M NaOH and 0.4 M Na_2_SO_3_ and reacted at 110 °C for 24 h. Balsam wood was then added to 1 wt% NaClO_2_ and the pH was adjusted to about 4.5 using acetic acid; the reaction was carried out at 90 °C for 24 h. Finally, the wood aerogel was washed several times with ethanol and distilled water to remove the chemicals, and the pre-frozen balsam wood was lyophilized in a freeze dryer for 24 h.

Then, anhydrous ferric chloride (1.6 g) and 40 mL of dichloromethane were added to a 250 mL double-necked flask and thoroughly stirred to disperse. In addition, 3CZ-TPA (0.305 g) was added to dichloromethane (20 mL) to form a solution, and wood aerogel (500 mg) was added and stirred thoroughly for 20 min. The treated wood aerogel was added to the anhydrous ferric chloride dispersion, and the reaction was stirred for 24 h under nitrogen at 25 °C (the molar ratio of the reactant 3CZ-TPA to ferric chloride was 1:24). After the reaction was completed, a certain amount of methanol was added to precipitate the solid, it was filter and it was extracted with methanol and tetrahydrofuran Soxhlet for 24 h to obtain CPOP/wood aerogel.

### 2.4. Adsorption-Photocatalysis Measurement

Firstly, the adsorption activity of the polymer RhB was characterized. The synthesized 2 mg of CPOP (0.04 mg/mL), wood aerogel or CPOP/wood aerogel was dispersed in 20 mg/L 50 mL of (RhB) solution. At different time periods (0, 1, 5, 8, 12, 20, 30, 40, 50, 60, 75 and 90 min), 5 mL of the solution was centrifuged at 7000 r/min for 5 min and the supernatant was filtered. In addition, the photocatalytic activity of the polymer in degrading RhB was characterized. For the degradation of RhB, the synthesized 2 mg of CPOP, wood aerogel or CPOP/wood aerogel was dispersed in 50 mL of 28 mg/L RhB solution and stirred for 24 h in the dark to reach adsorption–desorption equilibrium. Then, the solution was irradiated with a 60 W ultraviolet lamp, and the decomposed RhB solution was periodically extracted every 5 min (0–60 min). The absorption spectrum of RhB was measured with a UV-vis spectrophotometer (the characteristic absorption peak of RhB is located at 554 nm).

## 3. Results

### 3.1. Characterization of Materials

#### 3.1.1. Characterization of 3CZ-TPA

The successful synthesis of 3CZ-TPA was verified by ^1^H-NMR and ^13^C-NMR combined with mass spectrometry (Figures S1–S4). In this experiment, 3CZ-TPA was prepared by the Ullman reaction as shown in Figure S2. Chemical shifts of 1.56 ppm and 5.3 ppm are the peaks of a small amount of water and dichloromethane, and 7.26 ppm corresponds to the solvent peak of the deuterated chloroform used. Chemical shift peaks appear at 7.31 ppm, 7.46 ppm, 7.52 ppm, 7.54 ppm, 7.59 ppm and 8.18 ppm, which correspond to 6H at positions 5, 4, 1, 3, 2, and 6 on the benzene ring. The peak area ratio of these six peaks is 1:1:1:1:1:1, which corresponds to the type and number of hydrogens on 3CZ-TPA. In addition, Figure S3 is the mass spectrum of 3CZ-TPA. The calculated molecular weight of 3CZ-TPA is 740.29 g/mol, which is consistent with the results obtained from the mass spectrum. The ^13^C-NMR spectra of 3CZ-TPA exhibits characteristic peaks of 10 types of carbon atoms, corresponding to the structure of 3CZ-TPA (Figure S4). All of these results further verified the successful synthesis of 3CZ-TPA.

#### 3.1.2. Characterization of CPOP

**Figure 1:**
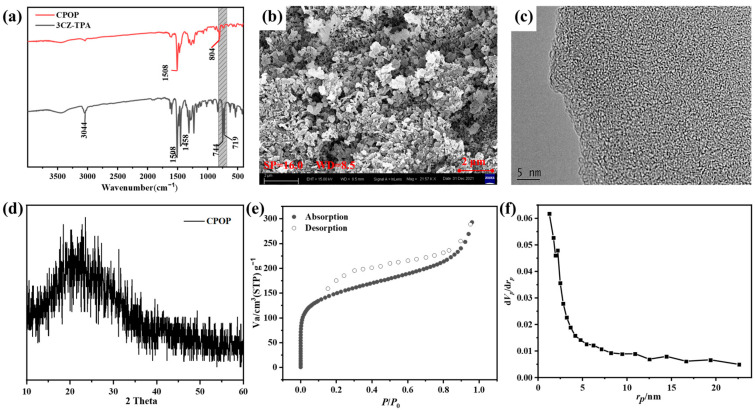
(**a**) FT-IR spectra of the monomer 3CZ-TPA and CPOP. (**b**) SEM image of CPOP. (**c**) TEM image of CPOP. (**d**) The X-ray diffraction (XRD) spectrum of CPOP. (**e**) Nitrogen sorption curves and the corresponding pore size distribution of CPOP. (**f**) Pore density profile.

The surface functional groups in CPOP and 3CZ-TPA were determined by FTIR spectra as shown in Figure 1a. CPOP and 3CZ-TPA showed a peak appearing at about 1508 cm^−1^ and 1458 cm^−1^ that is assigned to the stretchable vibration peaks of aromatic unsaturated C=C. The characteristic peak at 3044 cm^−1^ is the stretching vibration peak of aromatic unsaturated C-H in 3CZ-TPA. The peaks located at 744 cm^−1^ and 719 cm^−1^ are four adjacent hydrogen bond bending and vibration peaks on the benzene ring of the carbazole group. The characteristic peak of CPOP here is significantly smaller than that of 3CZ-TPA, which is attributed to the polymerization of carbazole. Additionally, CPOP shows a new peak at about 804 cm^−1^, which belongs to dimeric carbazole [29]. The peak intensity at 744 cm^−1^ and 719 cm^−1^ especially decreased significantly, and the production of new peaks indicated that CPOP had been successfully synthesized.

To investigate the morphology of the CPOP, SEM and TEM characterization were carried out (Figure 1b,c). The SEM image of CPOP showed a fused porous particle-like morphology, and there is an accumulation of irregular spherical nanoparticles. Micropores with uneven pore size and irregular void structure appear between the blocks. These structures increased the specific surface area of CPOP, which led to a larger contact area with the organic dye RhB in the adsorption process and photodegradation process, improving the photocatalytic ability [15,30]. The TEM images of CPOP show the characteristics of superposition, irregular particle shape, disordered accumulation and different thicknesses. This is due to the rigid structure presented by the long polymerization chain of CPOP, which has difficultly forming a close stacking, so the crystallinity is poor and the overall disordered stacking is presented. The X-ray diffraction (XRD) spectrum can reflect the arrangement of atoms inside the crystal. For CPOP, the broad peaks centered at 2*θ* = 21.5° are typically associated with the amorphous structure (Figure 1d) [18,31]. This corresponds to the results observed by SEM and TEM. The surface charge property of CPOP was determined by measuring the zeta potentials in the pH range of 2.0–12.0 (Figure S5). At pH = 7–8, the absolute value of Zeta potential exceeds 30, so the system is stable. When pH > 4.0, the superficial charge CPOP was negative. CPOP generates electrostatic attraction with the positively charged organic dye RhB, which is more conducive to the physical adsorption of RhB.

With a view to evaluating both the BET surface areas and the micro-porous nature of the CPOP, we applied depressed-temperature nitrogen adsorption–desorption isotherms. The CPOP was pretreated at 473 K for 180 min, and then the adsorption–desorption curve test was performed. As shown in Figure 1e, the typical isothermal curves obtained after analysis can be distinguished according to International Union of Pure and Applied Chemistry classification as isotherms with hysteresis of type H3 and with apparent hysteresis loops confirming the wide range of microporosity and mesoporosity of the produced CPOP. The adsorption of nitrogen by CPOP is significantly enhanced in the low-pressure part (P/P_0_ < 0.1), which showed an upward adsorption trend, demonstrating that there are many microporous structures in the polymer. Under the high-pressure section (P/P_0_ > 0.9) the adsorption capacity of the polymer increased significantly, probably due to the macropores generated by the particle packing. The specific surface area of CPOP was calculated by the Brunauer–Emmett–Teller theory, and could be as high as about 675 m^2^/g. This may be due to the strong rigidity of the conjugated structure of the polymer and the high content of benzene rings resulting in a void structure caused by steric hindrance [32]. The results of the pore density distribution of the polymer could be calculated by using non-local density functional theory (NLDFT) (Figure 1f), according to which CPOP contains many microporous structures with the main size of about 1 nm. These data suggested that the as-synthesized conjugated polymers possess a hierarchical pore structure, which can enhance mass transfer rates and facilitate dye particle penetration into CPOP, thereby enhancing their potential for adsorption application.

#### 3.1.3. Characterization of CPOP/Wood Aerogel

**Figure 2:**
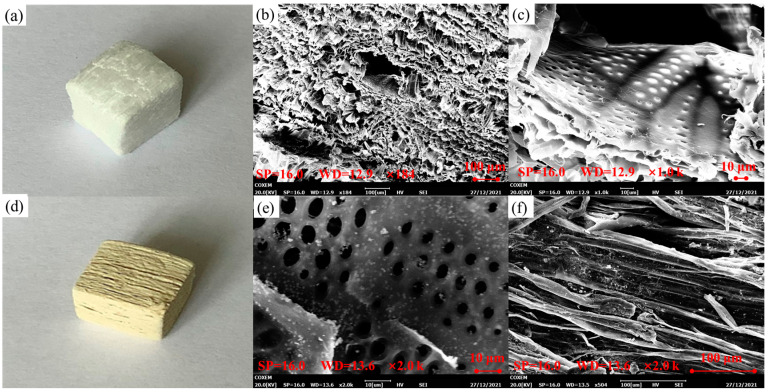
(**a**) The physical image of wood aerogel. (**b**) The transverse SEM of wood aerogel. (**c**) The longitudinal SEM of wood aerogel. (**d**) The physical image of CPOP/wood aerogel. (**e**,**f**) The longitudinal SEM of wood aerogel (magnification).

The morphology of wood aerogels and CPOP-loaded wood aerogels was analyzed by SEM. The wood aerogels, after the completion of the treatment, showed white color (Figure 2a), with cellulose structures uniformly arranged in their longitudinal direction. It can be seen from Figure 2b that, after removing the lignin and hemicellulose as fillers or carriers, the interior of the wood aerogel was uniformly arranged with a pore structure, and the size of the pore structure was about 70–100 μm in size. Figure 2c shows the lateral structure of wood aerogel, which inherits the 3D structure of natural wood and has the advantages of high porosity and high specific surface area; the pore diameter is about 10–60 μm. Further magnification of the void structure shows that there are still many small pores inside the void, indicating that the wood aerogel is a multi-dimensional network porous material.

Figure 2d is a physical photograph of the CPOP-loaded wood aerogel, whose appearance is pale yellow. There is still a good cellulose structure uniformly arranged in the longitudinal direction, and there is a good layered accumulation phenomenon in the transverse structure, which reflects the strong structural stability of wood aerogel. It can be seen from Figure 2e,f that a large amount of CPOP is dispersed inside the cavity of wood aerogel, especially on the three-dimensional network structure. Additionally, it can be seen that CPOP has good dispersion in wood aerogels, which indicates that CPOP is successfully loaded in wood aerogels.

### 3.2. Adsorption and Photocatalytic Performance

#### 3.2.1. Adsorption and Photocatalytic Performance of CPOP

**Figure 3:**
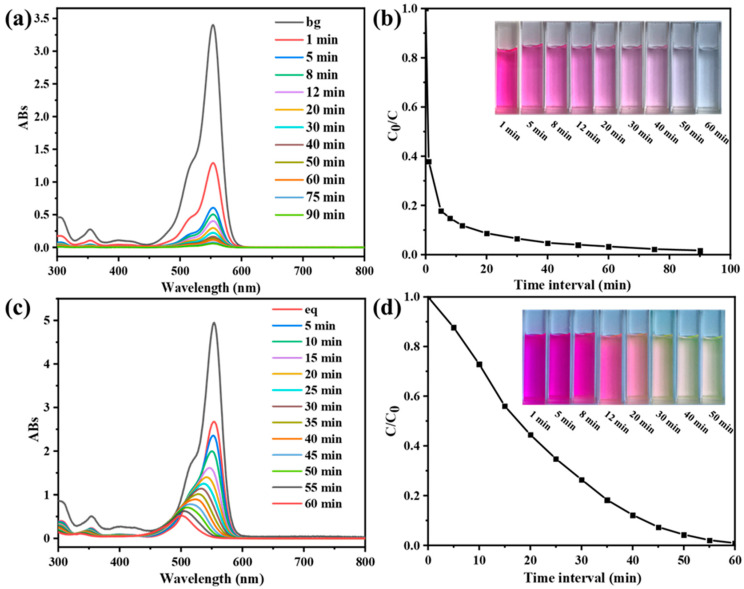
(**a**) UV-vis spectra of RhB (10 mg/L) at different time intervals in the presence of the compound CPOP (0.05 mg/mL) and in the absence of light. (**b**) Adsorption rate of CPOP (inset: color change of RhB during the process of adsorption). (**c**) UV-vis spectra of RhB at different time intervals in the presence of UV and the compound CPOP. (**d**) Photocatalytic rate diagram of CPOP (inset: color change of RhB during the process of degradation).

CPOP has the characteristics of high specific surface area, hierarchical pore structure etc. In addition, it has conjugated structure and high electron density at the nitrogen atom, which helps to adsorb small organic adsorbent, and can be used as an adsorbent to adsorb pollutants in water. The CPOP was ground and processed into powder, and the adsorption–desorption test of RhB was carried out under dark conditions; the results are shown in Figure 3. It can be clearly found from Figure 3a that the absorbance of the RhB characteristic peak (at 554 nm) gradually weakens as the absorption time continues to increase, and there is no movement of the characteristic peak. This shows that the content of RhB decreases and no new substances are formed in the adsorption–desorption process, which is attributed to physical changes. It can be seen from Figure 3b that the adsorption of CPOP to RhB is very fast in the first 5 min, which is caused by the microporous structure in CPOP, and dye absorption quickly reaches the plateau after 10 min, at which point 90% of the dye is adsorbed. The adsorption amount gradually decreases and the desorption amount increases gradually at 20–90 min, and finally it tends to the equilibrium state. CPOP completely adsorbs RhB in about 60 min, which also reflects that CPOP has a large specific surface area. During different adsorption–desorption time periods, the color of RhB gradually became lighter, from red to colorless, which indicated that it had good adsorption performance (Figure 3b, inset). The maximum adsorption capacity of RhB is 530 mg/g. From these results, CPOP is considered an outstanding candidate for removing RhB from water. In other words, a large number of carbazole building units showed excellent removal efficiency for water-soluble cationic organic dyes.

The photocatalytic ability of polymers can be improved by adjusting the energy band structure of polymers and expanding the absorption range of visible light wavelengths by heteroatom doping. In this experiment, the nitrogen atom was doped by introducing a carbazole group. Figure S6 shows the UV absorption spectra of CPOP, and the results show that the absorption of visible light of CPOP can be extended to 800 nm. By introducing a large amount of carbazole as a strong donor, the absorption range of the polymer becomes clearer towards the red region of the spectrum. This suggests that the HOMO-LUMO band gap decreases as more electron-donating parts are added to the polymer skeleton [33]. 

After CPOP reached the adsorption–desorption equilibrium in RhB solution, the photodegradation experiment was carried out by ultraviolet light irradiation. It can be seen from Figure 3c that the absorbance of RhB decreases continuously with the increase in time, and the characteristic peak was blue-shifted. This indicates that, during the photodegradation process, the concentration of RhB decreased continuously and new substances were produced, which is attributed to chemical changes. The RhB content in the solution changed from high to low within 60 min, as shown in Figure 3d. The degradation rate reached 99% at around 1 h, which reflects the excellent photodegradation ability of CPOP. The color of RhB gradually becomes lighter at different photodegradation times but still has color after degradation, which is the color of the new substance dissolved in water (Figure 3d, inset).

#### 3.2.2. Adsorption and Photocatalytic Performance of CPOP/Wood Aerogel

**Figure 4:**
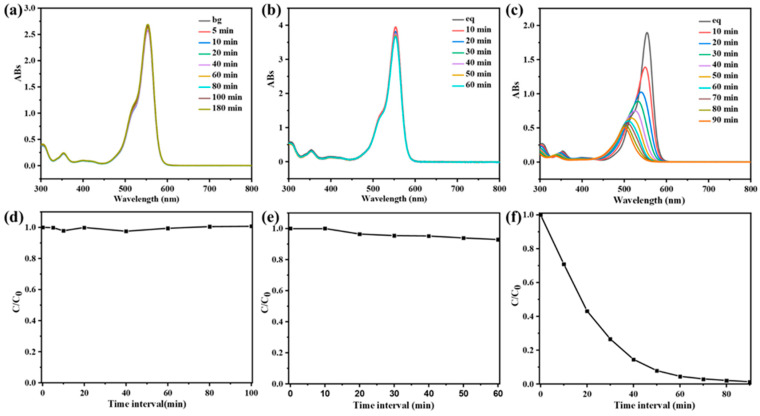
(**a**) UV-vis spectra of RhB at different time intervals in the presence of wood aerogel in the absence of light. (**b**) Adsorption rate of wood aerogel. (**c**) UV-vis spectra of RhB at different time intervals in the presence of UV and wood aerogel. (**d**) Photocatalytic rate diagram of wood aerogel. (**e**) UV-vis spectra of RhB at different time intervals in the presence of UV and CPOP/wood aerogel. (**f**) Photocatalytic rate diagram of CPOP/wood aerogel.

**Figure 5:**
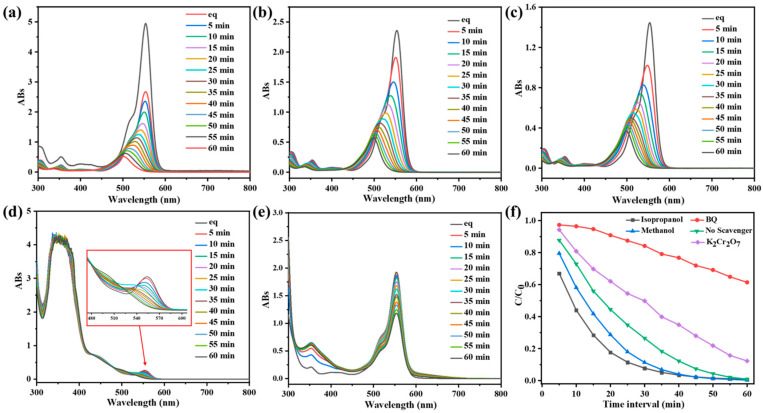
UV-vis spectra of RhB (10 mg/L) after different illumination time intervals in the presence of CPOP with different scavengers: (**a**) the blank control group, (**b**) methanol, (**c**) isopropanol, (**d**) potassium dichromate and (**e**) p-benzoquinone. (**f**) Effect of the different scavengers, the blank control group, methanol, isopropanol, potassium dichromate and p-benzoquinone, on the degradation of RhB over CPOP under 60 min of visible-light irradiation.

The wood aerogel was firstly added to the RhB solution, and the adsorption and photocatalytic degradation experiments were carried out. Figure 4a,b are the UV-vis spectra and process diagrams of wood aerogel adsorption–desorption. In Figure 5a, the absorbance does not change significantly with the increase of time, reflecting that wood aerogel has a small adsorption of RhB. Figure 4c,d are the UV-vis spectra and process diagrams of the photocatalytic degradation of wood aerogel. The absorbance of RhB hardly changes with the increase of the light time, which shows that the aerogel has almost no photocatalytic degradation ability for RhB. The unique 3D structure of wood aerogel was utilized as a carrier to load CPOP to obtain loaded aerogel to investigate its photocatalytic performance. The prepared CPOP/wood aerogel was added to the RhB solution, and Figure 4e,f are the UV-visible spectrum and process diagram of the photocatalytic degradation of CPOP/wood aerogel. It can be seen from the figure that, as the illumination time increases, the absorbance at the characteristic absorption wavelength of RhB decreases until it reaches zero. The photodegradation rate of RhB by CPOP/wood aerogel can reach more than 90% at 60 min, which is similar to the result for CPOP, indicating that both have strong photodegradation ability. After the degradation process, the CPOP/wood aerogel was collected and washed several times with methanol and deionized water and then dried, and the recovery was calculated to be about 90%. The comparison before and after loading shows that the high porosity and specific surface area of aerogels are used to increase the contact area between CPOP and RhB, which is beneficial to adsorption and catalysis, and they have good recyclability and reusability (Figure S7).

### 3.3. Photocatalytic Kinetics

To further understand the reaction kinetics involved in the degradation process of RhB by CPOP, pseudo-first-order and pseudo-second-order kinetic equations were used to perform fitting calculations on the reaction process, and the fitting equations were Formulas (1) and (2).

ln(C_0_/C) = k_1_ × t, (1)

1/C − 1/C_0_ = k_2_ × t, (2)

where k_1_ and k_2_ are pseudo-first-order and pseudo-second-order kinetic constants, the unit is min^−1^, and this expresses the degradation rate of photocatalytic dyes. t represents the time of illumination. C is the concentration of RhB solution at a certain moment of photodegradation. C_0_ represents the concentration of RhB solution after the CPOP reaches the adsorption–desorption equilibrium, the initial concentration of photodegradation. Fitted by the first-order kinetic equation, as shown in Figure S8a, the linear equation of CPOP degradation of RhB is y = 0.0778x − 0.7002, R^2^ = 0.9411. In the second-order reaction kinetics fitting diagram shown in Figure S8b, the linear equation for the degradation of RhB by CPOP is y = 0.4847x − 9.1895, R^2^ = 0.4791. R^2^ analysis and comparison showed that the process of CPOP degrading RhB was more consistent with the pseudo-first-order kinetic model. CPOP showed high catalytic efficiency in the photodegradation of RhB with a rate constant of 7.886 × 10^−2^ min^−1^ [34].

### 3.4. Photocatalytic Degradation Mechanism of CPOP

When the width of CPOP’s own energy band is less than or equal to the energy of the photon it absorbs, the photoelectrons in CPOP can jump from the valence band to the conduction band with high energy [35]. Therefore, CPOP generates electrons in the conduction band and holes in the valence band when excited by photons, resulting in an oxidation reaction capability. The generated photogenerated holes will come into contact with water and oxygen in water to generate hydroxyl radicals and peroxyl radical anions during photon excitation. Therefore, this experiment studied the role of electrons, holes, peroxyl radicals, hydroxyl radicals and singlet oxygen in the photocatalytic reaction process. A series of different capture agents were added to the CPOP degradation RhB solution to explore the effect of different capture agents, where no trapping agent was added as a blank control group (Figure 5a). Methanol was used as a hole trap and electron trap (Figure 5b), isopropanol as a hydroxyl radical trap (Figure 5c), potassium dichromate as an electron trap (Figure 5d), and p-benzoquinone (BQ) as a trap for peroxyl radicals (Figure 5e). 

As shown in Figure 5f, the addition of methanol and isopropanol did not affect the rate of RhB degradation by CPOP, indicating that holes and hydroxyl radicals did not play much role in the degradation process. However, the addition of potassium dichromate and BQ to the photodegradation system slowed down the rate of RhB degradation and the degradation efficiency became lower. This series of experimental phenomena shows that peroxy radicals play a vital role in the photocatalytic process, and the electrons generated on the conduction band also have a great influence on the photocatalytic process. According to Figure 4a, it can be concluded that the energy band structure of CPOP is relatively wide, and the generated electrons can capture oxygen to generate peroxyl radicals more strongly under the effect of light, which enhances its redox ability. In addition, the generated peroxy radicals can further oxidize and degrade organic dyes into small molecules, avoiding the recombination of generated electrons and holes, thereby enhancing the photocatalytic effect of CPOP.

### 3.5. Antibacterial Properties of CPOP

**Figure 6:**
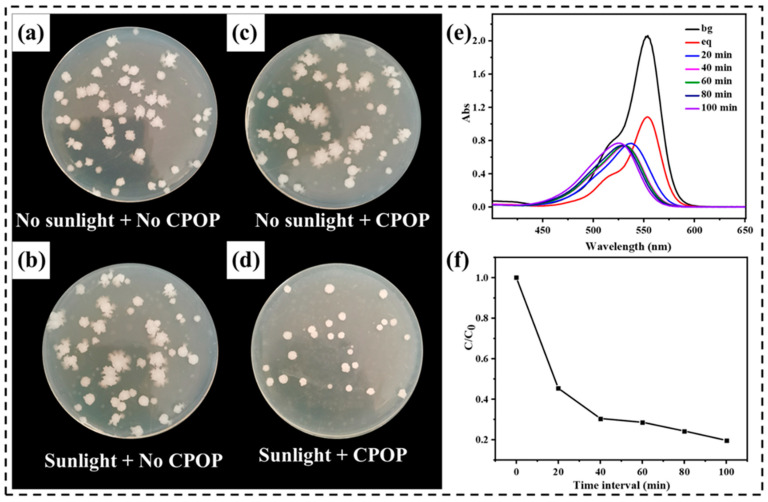
Photographs of *E. coli* and on agar plate as a control group in the absence of CPOP and light irradiation (**a**); the absence of CPOP + visible light irradiation (**b**); treated with CPOP in the dark (**c**); and visible light irradiation after CPOP treatment (**d**). (**e**) UV-vis spectra of RhB at different time intervals in the presence of wood aerogel in the sunlight. (**f**) Adsorption rate of wood aerogel in the sunlight.

In recent years, antimicrobial photodynamic therapy (PDT) has shown great promise as an alternative method for bacterial disinfection. Compared with traditional inactivation methods, PDT has the advantage of not inducing resistant strain selection because its mode of action is based on the production of reactive oxygen species (ROS) by light irradiation. Based on the excellent physicochemical stability, unique functional group, photocatalytic activity and peroxy free radical action of CPOP, the prepared polymer should be a good antibacterial agent [33]. The visible-light-promoted antibacterial disinfection ability of the CPOP was evaluated using Escherichia coli K-12 as Gram negative model systems. In this study, a suspension of bacteria was incubated with CPOP and exposed to sunshine light irradiation. As a note, all CPOP showed no toxic effects on bacteria cells in the absence of sunshine irradiation as shown in the dark experiment (Figure 6a,b). As shown under the same incubation conditions and concentration, the number of E. coli in the sunlight + CPOP was the lowest, indicating that its antibacterial activity was the highest, and CPOP was an effective antibacterial agent (Figure 6d). Figure 6c shows that CPOP itself has excellent biocompatibility, and its antibacterial activity is activated by photocatalysis to produce peroxy free radicals. Therefore, the active sites in the CPOP network and its inherent physicochemical properties are the reasons for its excellent antibacterial activity, which further broadens its application field and value.

### 3.6. Photocatalytic Degradation Performance under Actual Sunlight

In order to further verify the application of CPOP in real life, the photocatalytic degradation of RhB by CPOP was explored by replacing 60 W of UV light with sunlight (27 April 2022, Harbin Heilongjiang Province), and other conditions remained unchanged. It can be seen from Figure 6e that the absorbance at the characteristic peak of RhB decreases continuously with the increase of the illumination time, and the blue shift of the peak occurs. This indicates that the content of RhB decreased continuously and new substances were produced during this process. Figure 6f shows that the degradation rate of RhB is extremely fast within 0–40 min, and the degradation rate of RhB slows down to equilibrium within 40–100 min. This indicates that CPOP still has excellent photocatalytic degradation ability for RhB under sunlight irradiation, which has broad application prospects in dye removal.

## 4. Conclusions

In summary, we successfully synthesized CPOP from 3CZ-TPA containing N heteroatoms through oxidative coupling in this study. It possesses a high BET value (675 m^2^/g) and excellent catalytic activity. Additionally, this material also demonstrates excellent conventional physical adsorption (530 mg/g) and photodegradation capabilities (reaction rate constant of 7.886 × 10^−2^ min^−1^) for RhB organic dyes. Heteroatom doping broadens the light absorption range of CMPs to visible light, enabling successful photocatalysis under sunlight. CPOP was effectively loaded onto wood aerogels via in situ polymerization, addressing the issues of low solubility, poor dispersibility, and challenges in recycling and processing CMPs while enabling multi-cycle reuse. We used capture agents to explore the photocatalytic mechanism of CPOP. Peroxyl radicals play a crucial role in the photocatalytic process. Furthermore, the presence of peroxyl radicals endowed CPOP with an effective antibacterial function, resulting in CPOP have a self-cleaning capability.

## Data Availability

Not applicable.

## Supplementary Materials

Supporting Information can be downloaded at: https://www.mdpi.com/article/10.3390/polym15193929/s1.
