# Supplementary material for: A Conjugated Microporous Polymer/Wood Aerogel with Physical Adsorption, Chemical Degradation and Antibacterial Self-Cleaning Triple Sewage Treatment Functions
Source: Polymers (Basel). 2023 Sep 28;15(19):3929. doi: 10.3390/polym15193929 (PMC10574839; doi:10.3390/polym15193929)
Supplement: Supplementary file 1 [file polymers-15-03929-s001.zip › polymers-2621773-supplementary.pdf]

polymers

Supplementary Information

A Conjugated Microporous Polymer/Wood Aerogel with Physical Adsorption, Chemical Degradation and Antibacterial Self-Cleaning Triple Sewage Treatment Functions

Fanwei Kong, Junkang Ge, Zihao Zhu, Chunxia Chen, Jinsong Peng, Xiaobai Li *, Bin Li * and Hongwei Ma *

College of Chemistry Chemical Engineering and Resource Utilization, Northeast Forestry University, Harbin 150040, China; m15662556965@163.com (F.K.); junkangge@mail.ustc.edu.cn (J.G.); zzh1997lt@163.com (Z.Z.); ccx1759@163.com (C.C.); jspeng1998@163.com (J.P.)

* Correspondence: lixiaobai2008@126.com (X.L.); libin82192699@nefu.edu.cn (B.L.); mahw@nefu.edu.cn (H.M.)

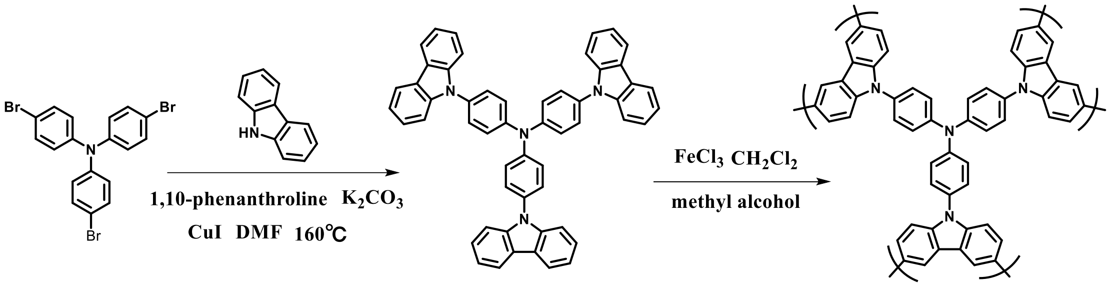

Scheme S1. Synthesis of 3CZ-TPA and CPOP.

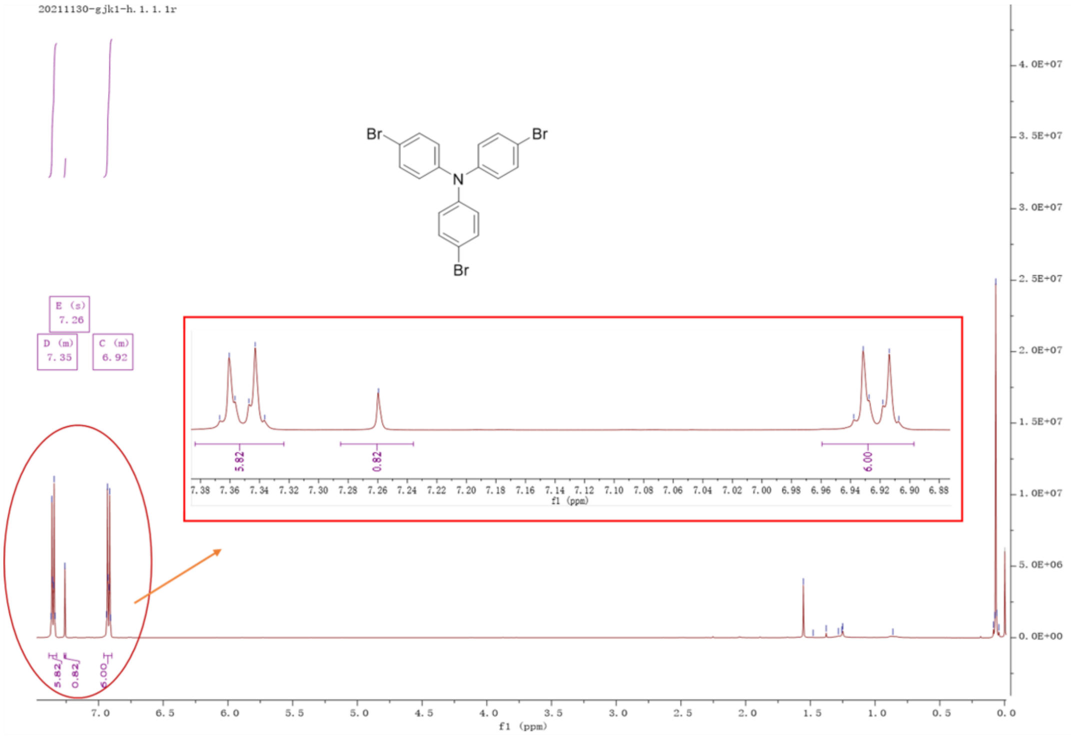

Figure S1. ^{1}H NMR spectra of tris(4-bromophenyl)amine.

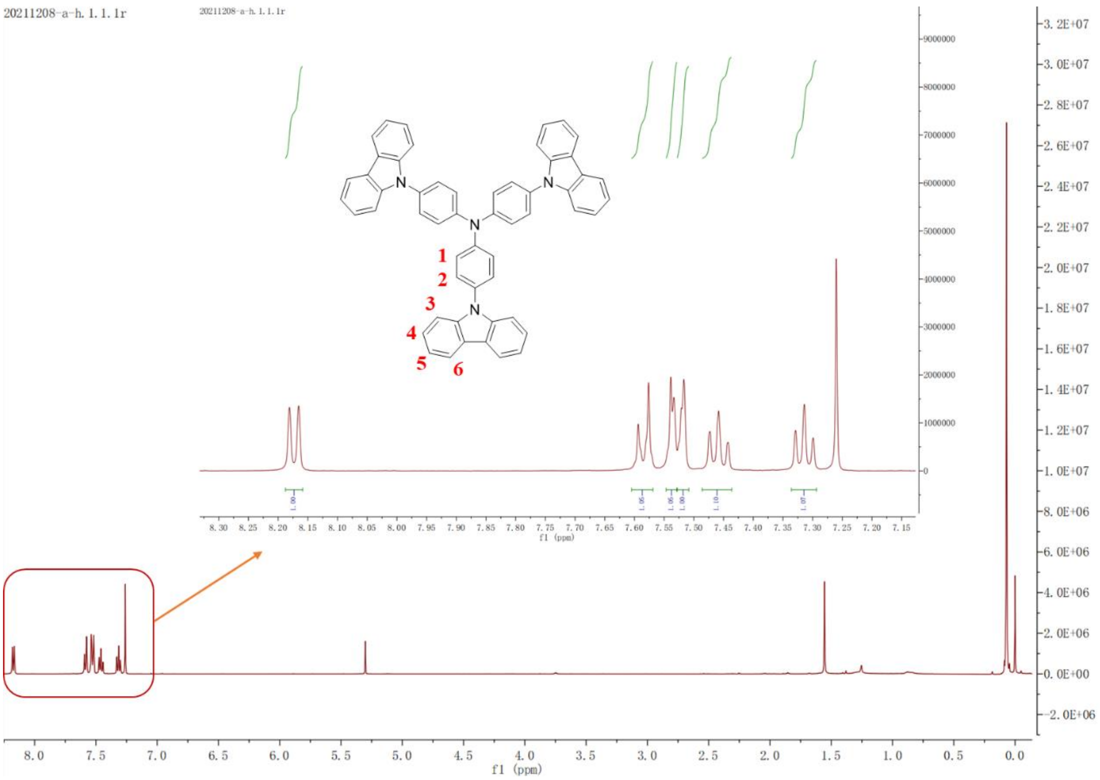

Figure S2. ^{1}H NMR spectra of tris(4-carbazolylphenyl)amine.

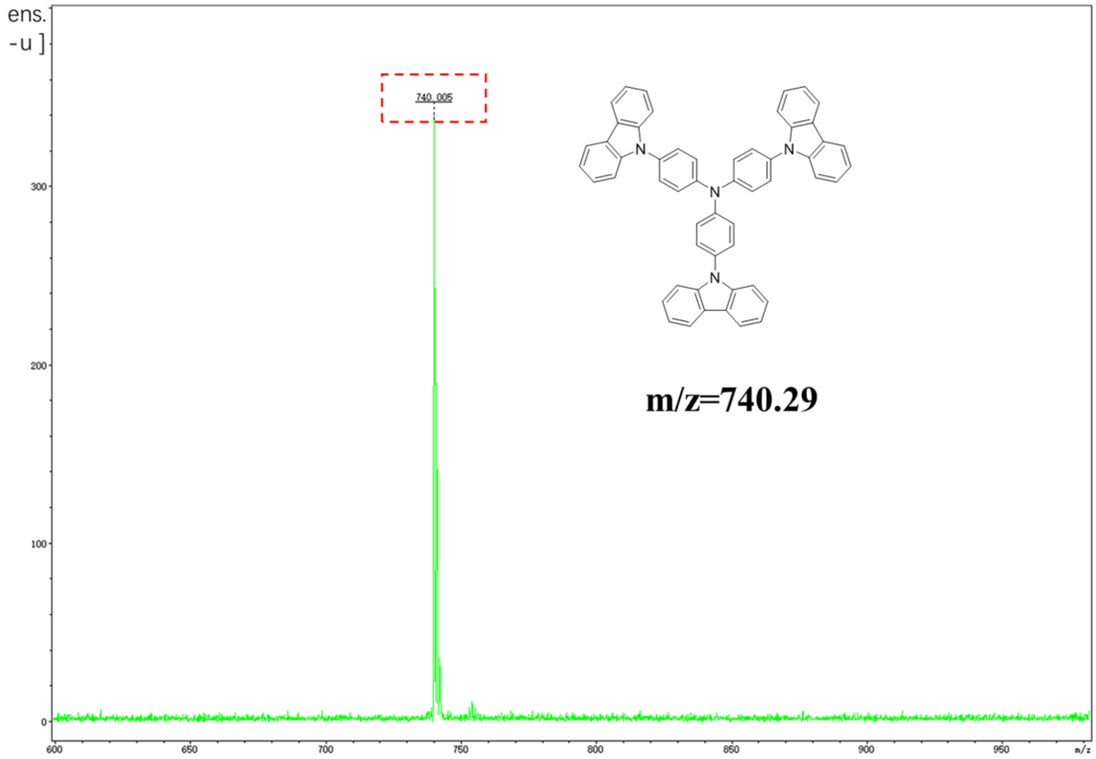

Figure S3. MALDI-TOF spectra of tris(4-carbazolylphenyl)amine.

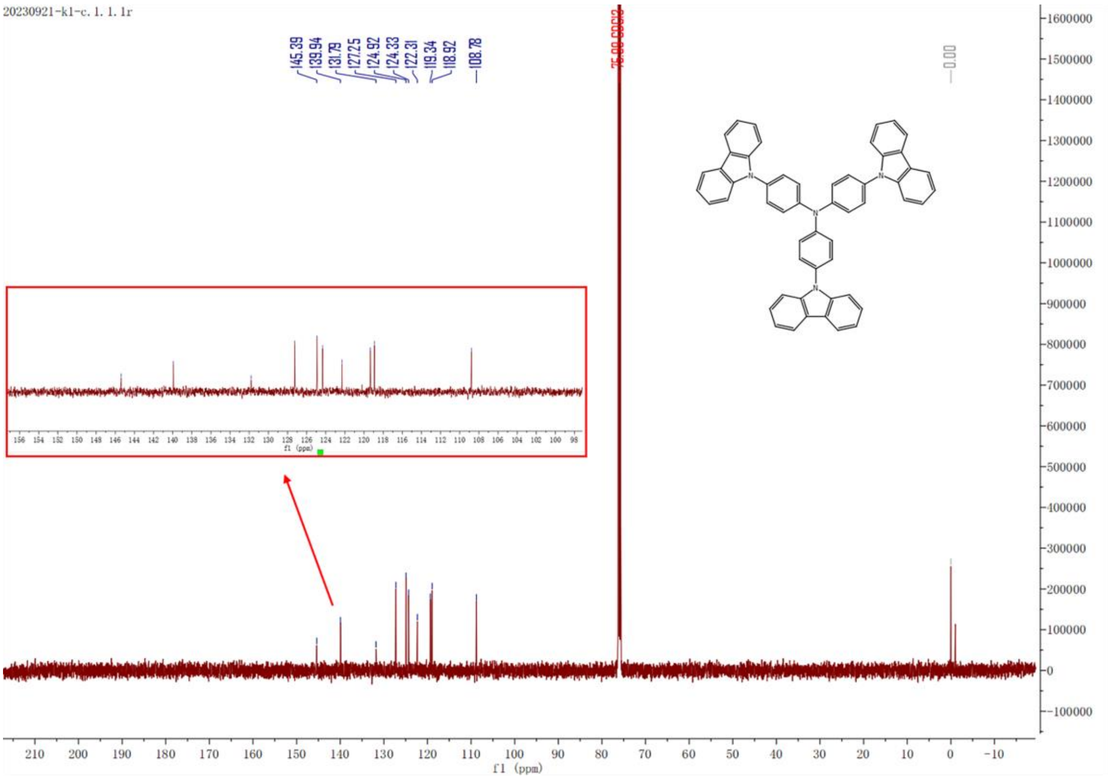

Figure S4. ^{13}C NMR spectra of tris(4-carbazolylphenyl)amine.

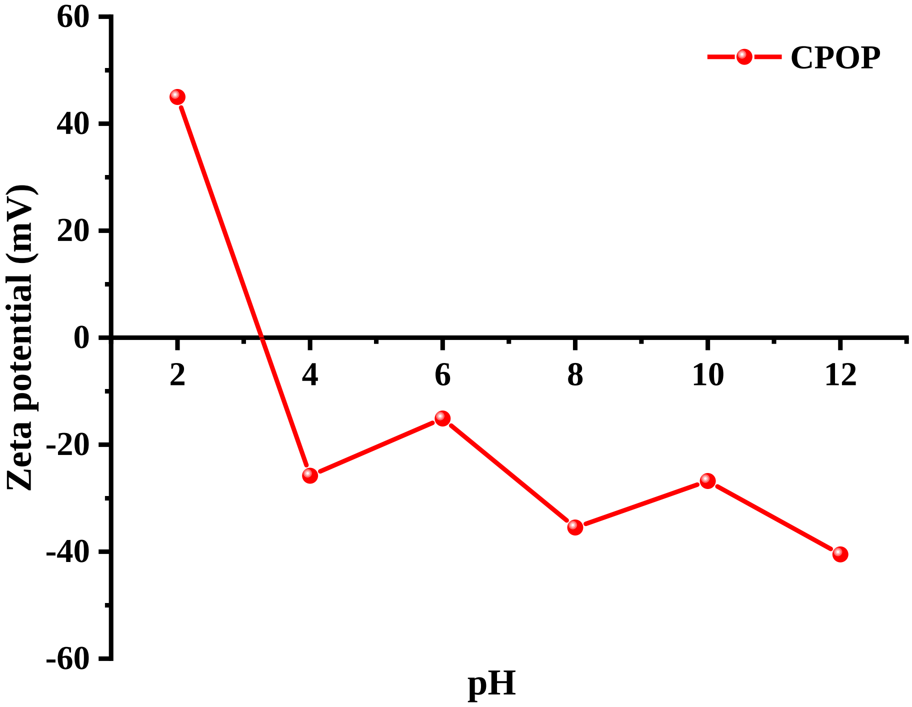

Figure S5. Zeta-potential of CPOP.

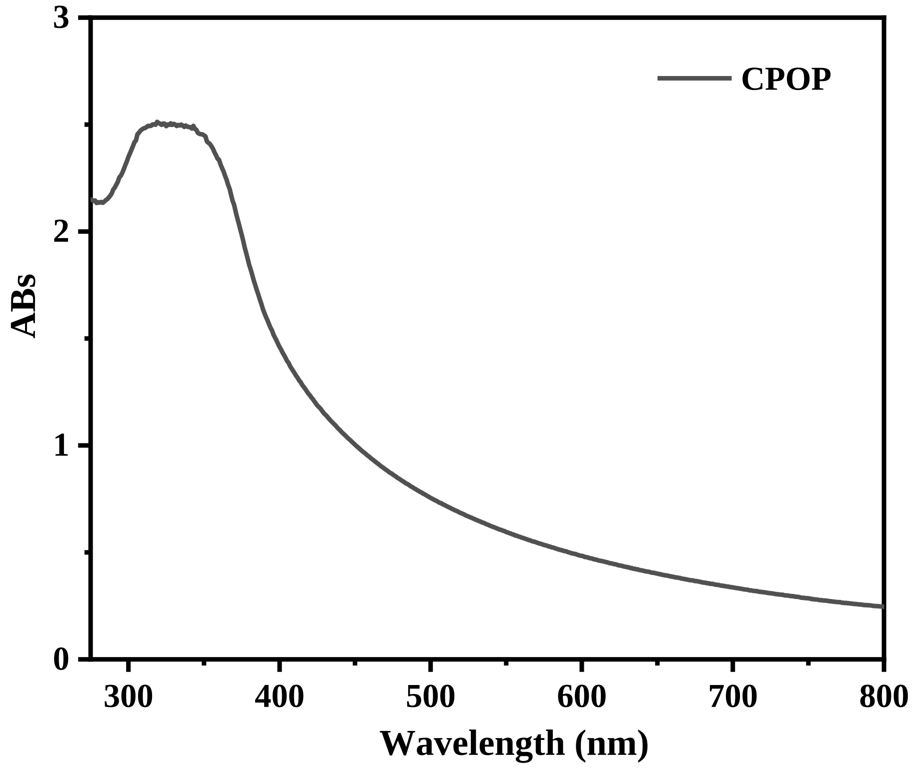

Figure S6. UV-vis spectra of CPOP.

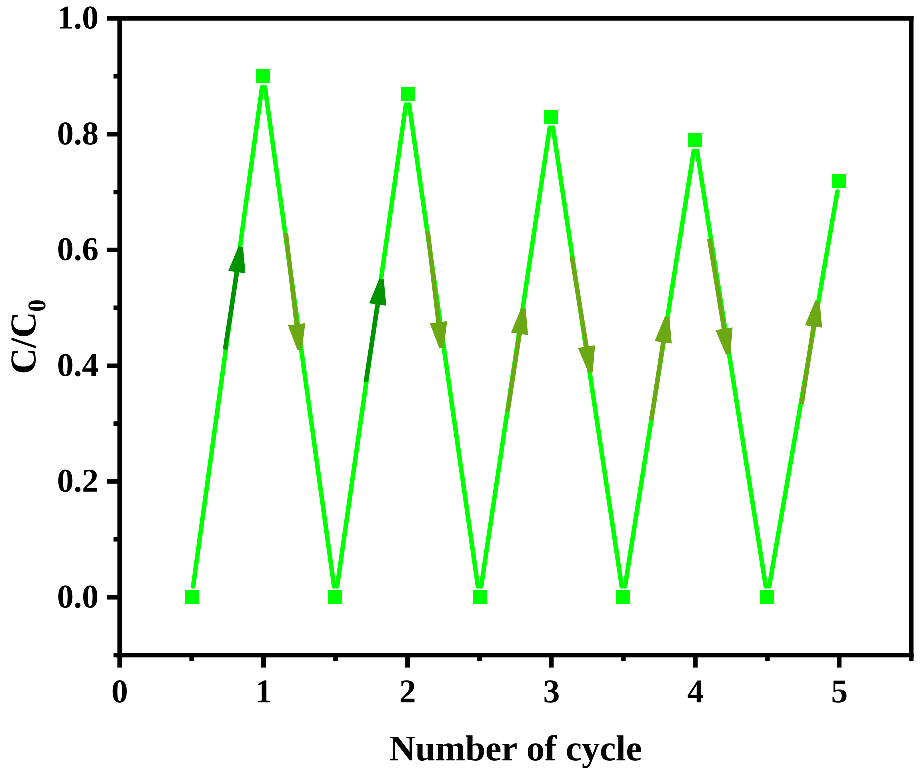

Figure S7. Cyclic stability of CPOP/wood aerogel.

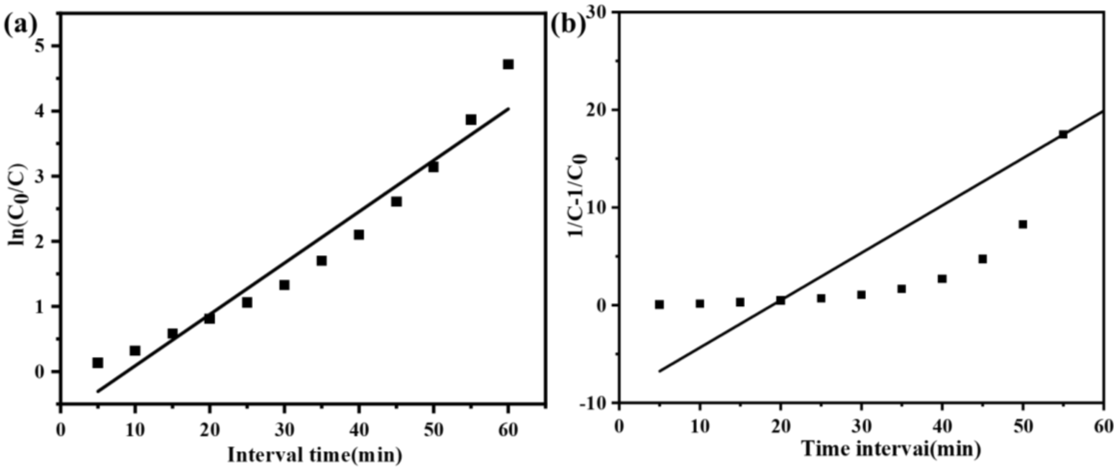

Figure S8. (a) Quasi-first-order dynamics of CPOP. (b) Quasi-second-order dynamics of CPOP.

Table S1. Comparison table of adsorption and photocatalytic performance between this work and other related works.

Materials	Adsorption	Concentration of catalysts	Concentration of RhB	Degradation Rate (over 80%)	References
PTZCP (CMP)	373 mg/g	0.05 mg/mL	10 mg/L	60 min	[1]
TiO_2	—	1 mg/mL	10 mg/L	180 min	[2]
BBO-NiPor-CMP	—	0.26 mg/mL	25 mg/L	80 min	[3]
CPOP	530 mg/g	0.04 mg/mL	8 mg/L	60 min	This work

References

1. Huang, Q.; Guo, L.; Wang, N.; Zhu, X.; Jin, S.; Tan, B. Layered Thiazolo[5,4-d] Thiazole-Linked Conjugated Microporous Polymers with Heteroatom Adoption for Efficient Photocatalysis Application. *ACS Appl. Mater. Interfaces.* **2019**, *11*, 15861-15868. http://dx.doi.org/10.1021/acsami.8b21765.
2. Chen, C.; Zhao, W.; Lei, P.; Zhao, J.; Serpone, N. Photosensitized Degradation of Dyes in Polyoxometalate Solutions Versus TiO2 Dispersions under Visible-Light Irradiation: Mechanistic Implications. *Chem. Eur. J.* **2004**, *10*, 1956-1965. https://doi.org/10.1002/chem.200305453.
3. Li, Y.; Duan, Q.; Wang, H.; Gao, B.; Qiu, N.; Li, Y. Construction of two-dimensional porphyrin-based fully conjugated microporous polymers as highly efficient photocatalysts. *J. Photochem. Photobiol. A.* **2018**, *356*, 370-378. https://doi.org/10.1016/j.jphotochem.2018.01.016.

Disclaimer/Publisher's Note: The statements, opinions and data contained in all publications are solely those of the individual author(s) and contributor(s) and not of MDPI and/or the editor(s). MDPI and/or the editor(s) disclaim responsibility for any injury to people or property resulting from any ideas, methods, instructions or products referred to in the content.
